# Ocular Phenotyping of Knockout Mice Identifies Genes Associated With Late Adult Retinal Phenotypes

**DOI:** 10.1167/iovs.66.6.64

**Published:** 2025-06-23

**Authors:** Abraham Hang, Andy Shao, Michael Shea, Michel J. Roux, Denise M. Imai-Leonard, David J. Adams, Takanori Amano, Oana V. Amarie, Zorana Berberovic, Raphaël Bour, Lynette Bower, Brian C. Leonard, Steve D. Brown, Soo Young Cho, Sharon Clementson-Mobbs, Abigail J. D'Souza, Mary Dickinson, Mohammad Eskandarian, Ann M. Flenniken, Helmut Fuchs, Valerie Gailus-Durner, Jason Heaney, Yann Hérault, Martin Hrabe de Angelis, Chih-Wei Hsu, Shundan Jin, Russell Joynson, Yeon Kyung Kang, Haerim Kim, Hiroshi Masuya, Ki-Hoan Nam, Hyuna Noh, Lauryl M. J. Nutter, Marcela Palkova, Jan Prochazka, Miles Joseph Raishbrook, Fabrice Riet, Jason Salazar, John Richard Seavitt, Radislav Sedlacek, Mohammed Selloum, Kyoung Yul Seo, Je Kyung Seong, Hae-Sol Shin, Toshihiko Shiroishi, Tania Sorg, Michelle Stewart, Masaru Tamura, Heather Tolentino, Uchechukwu Udensi, Sara Wells, Wolfgang Wurst, Atsushi Yoshiki, Hamid Meziane, Glenn Yiu, Paul A. Sieving, Louise Lanoue, K C. Kent Lloyd, Colin McKerlie, Ala Moshiri, for the International Mouse Phenotyping Consortium (IMPC)

**Affiliations:** 1 Department of Ophthalmology and Vision Science, University of California Davis Eye Center, Sacramento, California, United States; 2 Université de Strasbourg, CNRS (UMR 7104), Inserm (UMR-S 1258), Illkirch, France; 3 Department of Pathology, Microbiology and Immunology, School of Veterinary Medicine, University of California Davis, Sacramento, California, United States; 4 The Wellcome Trust Sanger Institute, Wellcome Genome Campus, Hinxton, Cambridge, United Kingdom; 5 RIKEN BioResource Research Center, Tsukuba, Japan; 6 Institute of Experimental Genetics, German Mouse Clinic, Helmholtz Zentrum München, Neuherberg, Germany; 7 The Centre for Phenogenomics, Lunenfeld-Tanenbaum Research Institute, Mount Sinai Hospital, Toronto, Ontario, Canada; 8 Université de Strasbourg, CNRS, INSERM, CELPHEDIA, PHENOMIN-Institut Clinique de la Souris (ICS), Illkirch-Graffenstaden, France; 9 Mouse Biology Program, University of California Davis, Davis, California, United States; 10 Department of Surgical and Radiological Sciences, School of Veterinary Medicine, University of California, Davis, Davis, California, United States; 11 Medical Research Council, Harwell Institute, Harwell, United Kingdom; 12 Department of Molecular and Life Science, Hanyang University, Seoul, Republic of Korea; 13 Mary Lyon Centre, Medical Research Council, Harwell Institute, Harwell, United Kingdom; 14 Department of Integrative Physiology, Baylor College of Medicine, Houston, Texas, United States; 15 Department of Molecular and Human Genetics, Baylor College of Medicine, Houston, Texas, United States; 16 Chair of Experimental Genetics, TUM School of Life Sciences, Technische Universität München, Freising, Germany; 17 German Center for Diabetes Research (DZD), Neuherberg, Germany; 18 College of Veterinary Medicine, Seoul National University, Seoul, Republic of Korea; 19 Laboratory Animal Center, Korea Research Institute of Bioscience and Biotechnology, Daejeon, Republic of Korea; 20 The Centre for Phenogenomics, The Hospital for Sick Children, Toronto, Ontario, Canada; 21 Czech Centre for Phenogenomics, Institute of Molecular Genetics of the Czech Academy of Sciences, Vestec, Czech Republic; 22 Department of Ophthalmology, Institute of Vision Research, Yonsei University College of Medicine, Seoul, Republic of Korea; 23 Laboratory of Developmental Biology and Genomics, Research Institute of Veterinary Science, BK21 Plus Program for Advanced Veterinary Science, College of Veterinary Medicine and Interdisciplinary Program for Bioinformatics, Seoul National University, Seoul, Republic of Korea; 24 Institute of Developmental Genetics, Helmholtz Munich, Neuherberg, Germany; 25 Developmental Genetics, Munich School of Life Sciences Weihenstephan, Technical University of Munich, Freising, Germany; 26 Munich Cluster of Systems Neurology (SyNergy), Munich, Germany; 27 German Center for Neurodegenerative Diseases (DZNE), Munich, Germany; 28 German Center for Mental Health (DZPG), Munich-Augsburg, Germany; 29 Department of Surgery, School of Medicine, University of California Davis, Sacramento, California, United States; 30 Department of Laboratory Medicine and Pathobiology, Faculty of Medicine, University of Toronto, Toronto, Ontario, Canada

**Keywords:** inherited retinal disease, knockout animals, age-related macular degeneration

## Abstract

**Purpose:**

Analyze phenotypic data from knockout mice with late-adult retinal pathologic phenotypes to identify genes associated with development of adult-onset retinal diseases.

**Methods:**

The International Mouse Phenotyping Consortium (IMPC) database was queried for genes associated with abnormal retinal phenotypes in the late-adult knockout mouse pipeline (49–80 weeks postnatal age). We identified human orthologs and performed protein–protein analysis and biological pathways analysis with known inherited retinal disease (IRD) and age-related macular degeneration (AMD) genes using Search Tool for the Retrieval of Interacting Genes/Proteins (STRING), PLatform for Analysis of single cell Eye in a Disk (PLAE), Protein Analysis Through Evolutionary Relationships (PANTHER), and Kyoto Encyclopedia of Genes and Genomes (KEGG).

**Results:**

Screening of 587 late-adult mouse genes yielded 12 with abnormal retinal phenotypes, which corresponded to 20 human orthologs. Three of the 12 mouse genes and two of the 20 human orthologs were previously implicated in retinal pathology or physiology in a literature review. Although all of the genes demonstrated retinal pathology when deleted from the mouse genome, most do not have established roles in human retinal disease. Furthermore, human protein–protein analysis and biological pathway analysis yielded only a few relationships between the candidate gene list and that of known IRD and AMD genes, suggesting they may represent novel retinal functions.

**Conclusions:**

We identified 12 mouse genes with significant late-adult abnormal retinal pathology, eight of which have not been previously implicated in either mouse or human retinal physiology or pathology. These serve as novel retinal disease gene candidates for late-onset retinal disease.

The genetic basis of retinal biology and photoreceptor function is a fast-moving field of active research. Despite a large body of literature arising from many scientific groups around the globe over the past several decades, the biology of photoreceptors and other retinal cells is incompletely understood. This field of research is relevant to single gene disorders of the retina in human populations. Inherited retinal diseases (IRDs) are a heterogeneous group of disorders, both phenotypically and genetically, which result in degeneration of the retina and vision impairment. The estimated prevalence of IRDs is around 1 in every 2000 to 3000 individuals,^1^^,^^2^ and they are predominantly monogenic in etiology, causing photoreceptor degeneration or dysfunction. There are currently around 300 genes that have been associated with IRDs.^3^^,^^4^ Genetic testing can provide a definitive diagnosis and help provide prognostic data for the patient as well as for at-risk family members. Multiple next-generation sequencing (NGS) panels are currently available, including comprehensive and disease-specific panels^5^; however, panel-based NGS still fails to diagnose 30% to 40% of IRDs.^6^^,^^7^

An incomplete understanding of genetics and the heterogeneity of clinical presentation make IRDs challenging to diagnose, especially because some individuals may have slow-onset disease or a clinical picture that may be confused with other diagnoses such as age-related macular degeneration (AMD) or resolved ocular inflammation. Therefore, the identification of new genes or biological pathways specifically implicated in late-onset IRD is necessary to distinguish phenotypically overlapping entities from one another. Elucidating these genetic underpinnings not only enhances our biological understanding but also improves the accuracy of clinical diagnosis.

Formed in 2011, the International Mouse Phenotyping Consortium (IMPC) is comprised of an international group of research institutions that produce single-gene knockout mouse lines using gene-targeted embryonic stem cells or, more recently, CRISPR/Cas9 methodology.^8^^,^^9^ The Consortium aims to complete the knockout resource for the human orthologous genome and provide broad-based phenotyping for each knockout line produced. At least 14 mice (a minimum of seven males and seven females), along with matched wild-type controls, are produced for each knockout line. When viable, homozygous mice are phenotyped; however, when homozygous mice are not viable, heterozygous mice are evaluated. All viable mice undergo a uniform regimented battery of phenotyping tests from 4 to 16 weeks postnatal age in the “early adult pipeline,” after which terminal procedures are performed. In a pilot project, 587 mouse strains were also phenotyped between 49 and 80 weeks in the “late-adult pipeline” to identify phenotypes present in the later adult period.

These late adult phenotypes are of great interest for retinal pathologies such as AMD or slow-onset IRDs, which only appear in the late-adult time frame and would not be observed during early adult phenotyping. Here, we report on 12 genes from knockout mouse phenotyping identified through a systematic query of the IMPC database that are associated with late-onset retinal pathology.

## Methods

### Mouse Background

Each IMPC center adheres to strict ethical review and licensing procedures, following both its regional regulatory guidelines as well as the Animal Research: Reporting of In Vivo Experiments (ARRIVE) guidelines, a checklist of items designed to increase transparency and provide standardization.^10^ A minimum of seven male and seven female knockout mice are generated, with standardized procedures performed with regard to phenotyping and eye examination procedures, including an anterior slit-lamp exam, iris/pupil response, and a dilated exam with imaging of abnormal findings as capacity permits. Several different strategies have been employed in generating knockout mice, with increasing use of CRISPR/Cas9. Other strategies that have been used include the “knockout-first” allele design using site-specific recombinases Cre and Flp, as well as the VelociGene null allele design to delete protein coding sequences of a target gene. Phenotyping is conducted at each center, and procedures are assessed routinely to ensure animal welfare.

### Statistical Analysis

Statistical analyses were conducted by the IMPC using the OpenStats toolkit in R (R Foundation for Statistical Computing, Vienna, Austria) to provide appropriate data analysis depending on the data type. Fisher's exact tests were used for categorical data, and abnormal phenotypes for a specific trait were compared collectively to the normal phenotype. The IMPC analysis does not take into account whether an abnormality is fully or incompletely penetrant, and the same statistical weight is given whether the abnormal phenotype is present in one or both eyes. Statistical significance was defined as *P* < 0.0001. If the phenotype was determined to be significant, it was further determined if this effect was the same and present in both sexes or in only one sex. In cases where the center was not able to determine if an abnormal phenotype was present, the mouse was excluded from the analysis.

### Data Query

The IMPC database was queried on Data Release 19.0. As of May 9, 2023, it contained 8491 phenotyped genes, 9138 phenotyped mutant lines, and 102,805 phenotype calls, including both early- and late-onset mutant lines. Of these, 587 mutant lines were phenotyped in the late-adult pipeline. Mouse lines with an abnormal retinal phenotype only in the late-adult pipeline were included in this study, whereas knockout lines with abnormal phenotypes in the early and late adult stages were excluded. After an initial automated search, each gene was manually reviewed to confirm an abnormal phenotype was identified. The Retinal Information Network (RetNet) database, which tracks genes causing inherited retinal diseases, was also queried to provide a list of known genes involved in retinal diseases. Additionally, a search was done through the National Human Genome Research Institute and European Bioinformatic Institute (NHGRI-EBI) genome-wide association study (GWAS) catalog to identify all known genes associated with AMD.

### Literature Search

A literature search was conducted on PubMed using the gene name as well as the search term “eye” and separately with the search term “retina,” and each article was reviewed for any reported association of the gene being involved in retinal pathophysiology.

### Bioinformatic Analysis

Human orthologs of all knockout mouse genes were identified using the GeneCards human gene database by entering the mouse gene and [ortholog] as the search term. The human ortholog was confirmed to match with the mouse gene if the identical name was found in the ortholog section of the queried gene. The human ortholog found set was then used for further analyses (Supplemental Materials). Protein Analysis Through Evolutionary Relationships (PANTHER) and Kyoto Encyclopedia of Genes and Genomes (KEGG) were used to identify biological pathways associated with the human ortholog found set. Search Tool for the Retrieval of Interacting Genes/Proteins (STRING) was used to identify protein–protein interactions between the human ortholog found set and the human RetNet and GWAS gene lists using a confidence cutoff of 0.7. The STRING network was expanded by adding secondary interacting proteins that were not initially identified with a selectivity of interactors of 0.7 to look for additional protein–protein interactions. PLatform for Analysis of single cell Eye in a Disk (PLAE), version 0.94, was used to evaluate gene expression of the human ortholog found set by retina cell type.^11^

### Histopathology

Complete necropsy was done, and all abnormal phenotypic findings were recorded using standardized IMPC Gross Pathology ontology.^12^ Tissue samples were then fixed, typically in 10% neutral buffered formalin, sectioned at 5 µm, stained with hematoxylin and eosin, and examined by a veterinary pathologist. Mild to moderate focal retinal dysplasia was considered incidental and attributed to the C57BL/6N substrain, which is known to be homozygous for the *rd8* mutation in the *Crb1* gene, causing background retinal abnormalities.^13^^,^^14^

### Fundus Photography

At imaging-capable centers, fundus photography was obtained to document fundus abnormalities. In general, mice were anesthetized with intraperitoneal ketamine/midazolam, and their eyes were dilated with topical tropicamide or topical tropicamide and phenylephrine, then lubricated with methylcellulose-containing artificial tears prior to fundus photographs being taken with a MICRON III or IV retinal imaging system (Phoenix-Micron, Inc., Bend, OR, USA). Techniques and protocols varied slightly among imaging centers. Although most centers performed screening using indirect ophthalmoscopy, the Baylor College of Medicine site also performed optical coherence tomography screening.

## Results

In total, 12 knockout mouse lines (Table) in the IMPC database had retinal pathology identified only in the late-adult pipeline, where phenotyping was extended from 49 to 80 weeks depending on the center in which the mice were examined. These were *Abhd17b*, *Atp8b1*, *Calcoco1*, *Gnb4*, *Mt1*, *Nt5dc1*, *Sorl1*, *Spink12*, *Tat*, *Thsd7a*, *Tmem51*, and *Trim39*.

**Table.** Knockout Mouse Genes With Abnormal Late Retinal Phenotype

Mouse Gene Symbol	Mouse Gene Name	Mouse Genome Informatics ID	Human Ortholog Symbol	Phenotype Summary
*Abhd17b*	Abhydrolase domain containing 17B	1917816	*ABHD17B*	Abnormal retina morphology
*Atp8b1*	ATPase, class I, type 8B, member 1	1859665	*ATP8B1*	Abnormal retinal blood vessel morphology
*Calcoco1*	Calcium binding and coiled coil domain 1	1914738	*CALCOCO1*	Abnormal retina morphology
*Gnb4*	Guanine nucleotide binding protein (G protein), beta 4	104581	*GNB4*	Abnormal retina morphology
*Mt1*	Metallothionein 1	97171	*MT1A*, *MT1B*, *MT1E*, *MT1F*, *MT1G*, *MT1H*, *MT1HL1*, *MT1M*, *MT1X*, *MT2A*	Abnormal retina inner nuclear layer morphology
*Nt5dc1*	5′-Nucleotidase domain containing 1	2442446	*NT5DC1*	Abnormal retina morphology
*Sorl1*	Sortilin-related receptor, LDLR class A repeats-containing	1202296	*SORL1*	Abnormal retina morphology
*Spink12*	Serine peptidase inhibitor, Kazal type 12	1925492	None	Abnormal retina morphology
*Tat*	Tyrosine aminotransferase	98487	*TAT*	Abnormal retina morphology, abnormal retina blood vessel morphology
*Thsd7a*	Thrombospondin, type I, domain containing 7A	2685683	*THSD7A*	Abnormal retina morphology
*Tmem51*	Transmembrane protein 51	2384874	*TMEM51*	Abnormal retina morphology
*Trim39*	Tripartite motif-containing 39	1890659	*TRIM39*	Abnormal retina morphology

The identified retinal phenotypes were abnormal retina morphology, abnormal retina inner nuclear layer morphology, and abnormal retina blood vessel/vasculature morphology. Only one zygosity was examined in these genes, with all lines being homozygous knockouts. Notably, many of these genes exhibited sexual dimorphism, with the retinal phenotype achieving significance in either males or females only. The knockout genes that achieved significance in females only were *Abhd17b*, *Calcoco1*, *Mt1*, and *Sorl1*. The knockout genes that achieved significance in males only were *Tat*, *Thsd7a*, *Tmem51*, *Trim39*, *Nt5dc1*, and *Spink12*, which are all on autosomal chromosomes.

**Figure 1.**
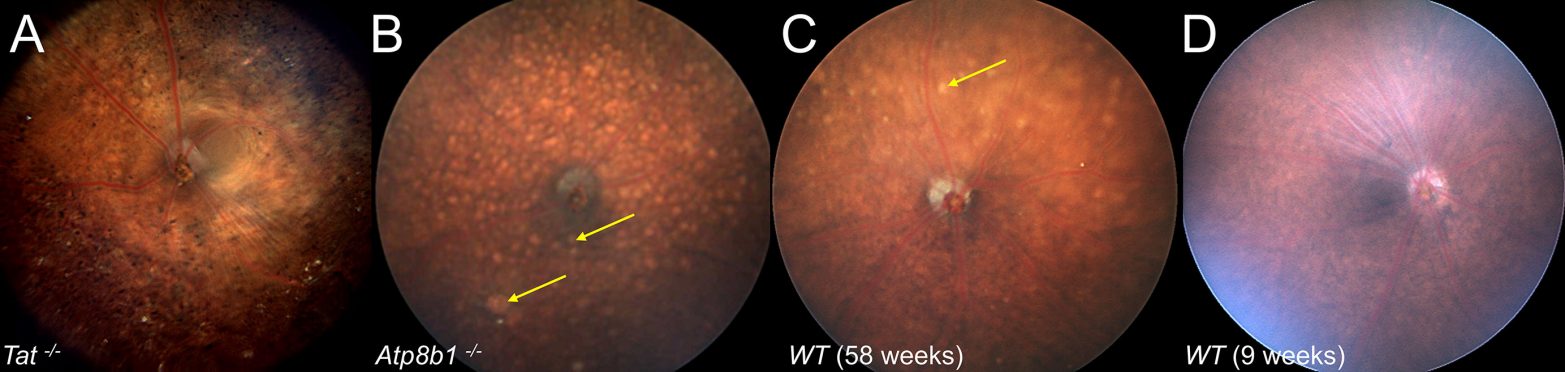
Fundus photograph examples of knockout lines with retinal abnormalities. (**A**) *Tat* homozygote at 75 weeks with pigmentary changes throughout the fundus. (**B**) *Atp8b1* homozygote at 58 weeks with numerous *yellow spots* and a lesion below the optic nerve (*arrow*). (**C**) Wild-type C57BL/6NCrl at 58 weeks for comparison. Some *yellow spots* are seen (*arrow*) consistent with background *rd8* mutation. (**D**) Wild-type C57BL/6NCrl at 9 weeks.

**Figure 2.**
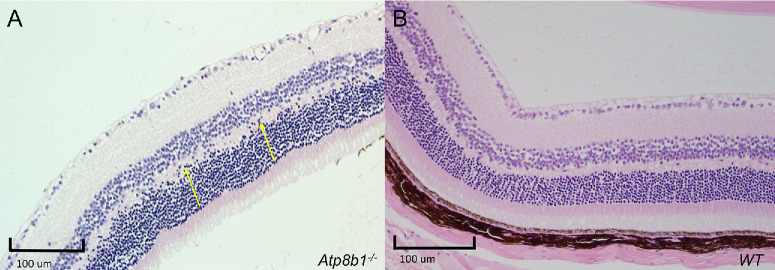
Histological example of knockout line with retinal abnormalities. (**A**) *Atp8b1* homozygote knockout at 59 weeks demonstrating numerous cells present in the outer plexiform layer (*yellow arrows*). (**B**) Wild-type at 59 weeks showing normal anatomy of the retina.

Examples of fundus photographs depicting abnormal retinal morphology (*Atp8b1* and *Tat*) are shown in Figure 1, as well as an example of abnormal retinal histology in Figure 2. As part of this study, further careful examination of *Atp8b1* was done retrospectively with the phenotyping site, revealing a subtle abnormal phenotype in the early adult stage that was initially missed. It is unclear if subtle abnormalities may have been identified in the early stage in any of the other 11 genes with careful retrospective examination that would be missed on prospective screening; given this possibility, the gene was included in this study.

**Figure 3.**
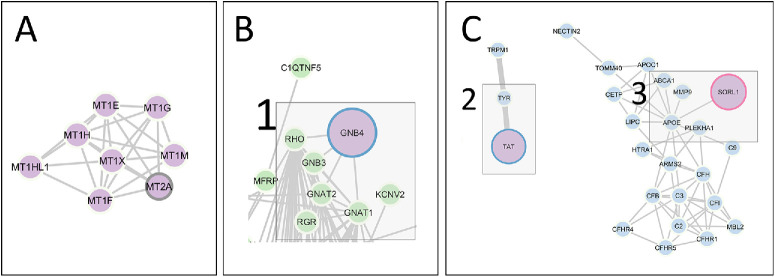
STRING analysis of protein–protein interactions. (**A**) Interaction between *MT1* candidate gene orthologs (*purple*). (**B**) Interaction between *GNB4* (1) (*purple*) with RetNet database genes (*green*). (**C**) Interaction between *TAT* (2) and *SORL1* (3) candidate genes (*purple*) with AMD GWAS genes (*blue*). Organism set to *Homo* *sapiens* (settings: network type = full STRING network, selectivity of interactors = 0.7). Nodes corresponding to genes for which there were PubMed reports of implication in retinal physiology or pathology in human, mouse or both are circled respectively in *gray*, *blue*, or *red*.

The set of 12 knockout mouse genes was converted to the corresponding 20 human orthologous genes, with one mouse gene (*Mt1*) having 10 human orthologs and another gene (*Spink12*) lacking a known human ortholog. A literature search of these 20 human orthologs revealed that two genes (*MT2A*and *SORL1*) have been previously associated with human retinal physiology or pathology,^15^^,^^16^ and three genes (*Sorl1*, *Tat*, and *Gnb4*) have been implicated in mouse retinal physiology or pathology.^17^^–^^19^ Notably, polymorphisms in *MT2A* have been associated with AMD in Northern Spanish patients.^16^ Eight mouse genes corresponding to seven genes in the human ortholog found set (*Spink12* has no known human ortholog) have not been previously associated with human retinal pathophysiology, nor have their corresponding mouse genes been linked to any significant retinal processes. STRING analysis with a selectivity of interactors of 0.7 was used to identify potential protein-protein interactions within the set of 20 human orthologs. No interactions were predicted (Supplementary Fig. S1), except for the human orthologs of *Mt1* as a self-interacting protein (Fig. 3A).

Each gene and its human ortholog were then queried in the PLAE ocular meta-atlas, a resource for single-cell transcriptomics from ocular cell types in several species. Of the 12 knockout mouse genes, eight (67%) were expressed in the retina (*Abhd17b*, *Calcoco1*, *Gnb4*, *Mt1*, *Sorl1*, *Thsd7a*, *Tmem51*, and *Trim39*). Seven (58%) were expressed in the retinal pigmented epithelium (RPE; *Abhd17b*, *Calcoco1*, *Mt1*, *Sorl1*, *Thsd7a*, *Tmem51*, and *Trim39*); all genes that were expressed in the RPE were also expressed in retina except *Gnb4*, which was only expressed in the retina and not the RPE. Of the 20 human orthologs, 14 (70%) were expressed in the retina and RPE (*Calcoco1*, *Gnb4*, *Mt1a*, *Mt1e*, *Mt1f*, *Mt1g*, *Mt1h*, *Mt1h*, *Mt1x*, *Mt2a*, *Nt5dc1*, *Sorl1*, *Thsd7a*, and *Tmem51*), and the remaining six were not expressed in either tissue. The expression of three genes (*Atp8b1*, *Tat*, and *Spink12*) was not detected in either mouse or human retina or RPE.

The 20 human orthologs were compared with 264 genes known to cause inherited retinal disease, including macular dystrophies and retinitis pigmentosa, using the RetNet database and analyzed using STRING. No large clusters of protein interactions were identified between the RetNet dataset and the human ortholog found set (Supplementary Fig. S2), even with an additional 20 closest interactors. Notably, even among the RetNet genes themselves, many were singletons and did not have known interactors within the database. There was one knockout gene that did interact with the RetNet dataset (Fig. 3B): *GNB4*, a constituent of heterotrimeric G proteins.

To determine if the genes identified in the late-adult pipeline may be involved in retinal diseases of aging, we used similar bioinformatic strategies to assess relationships with loci implicated in AMD. A list of 123 unique AMD-associated genes was obtained through the NHGRI-EBI GWAS catalog using AMD as the “trait.” STRING analysis was used to look for protein–protein interactions between the 20 human orthologs and these 123 GWAS AMD genes (Supplementary Fig. S3). No large clusters of protein interactions were identified, including when an additional 10 interactors were added. Two knockout genes interacted with the GWAS AMD genes: sortilin-related receptor 1 (*SORL1*), a gene coding for a receptor involved in intracellular sorting of protein which is primarily associated with Alzheimer disease,^20^ and tyrosine aminotransferase (*TAT*), which is involved in the tyrosine breakdown pathway (Fig. 3C).

**Figure 4.**
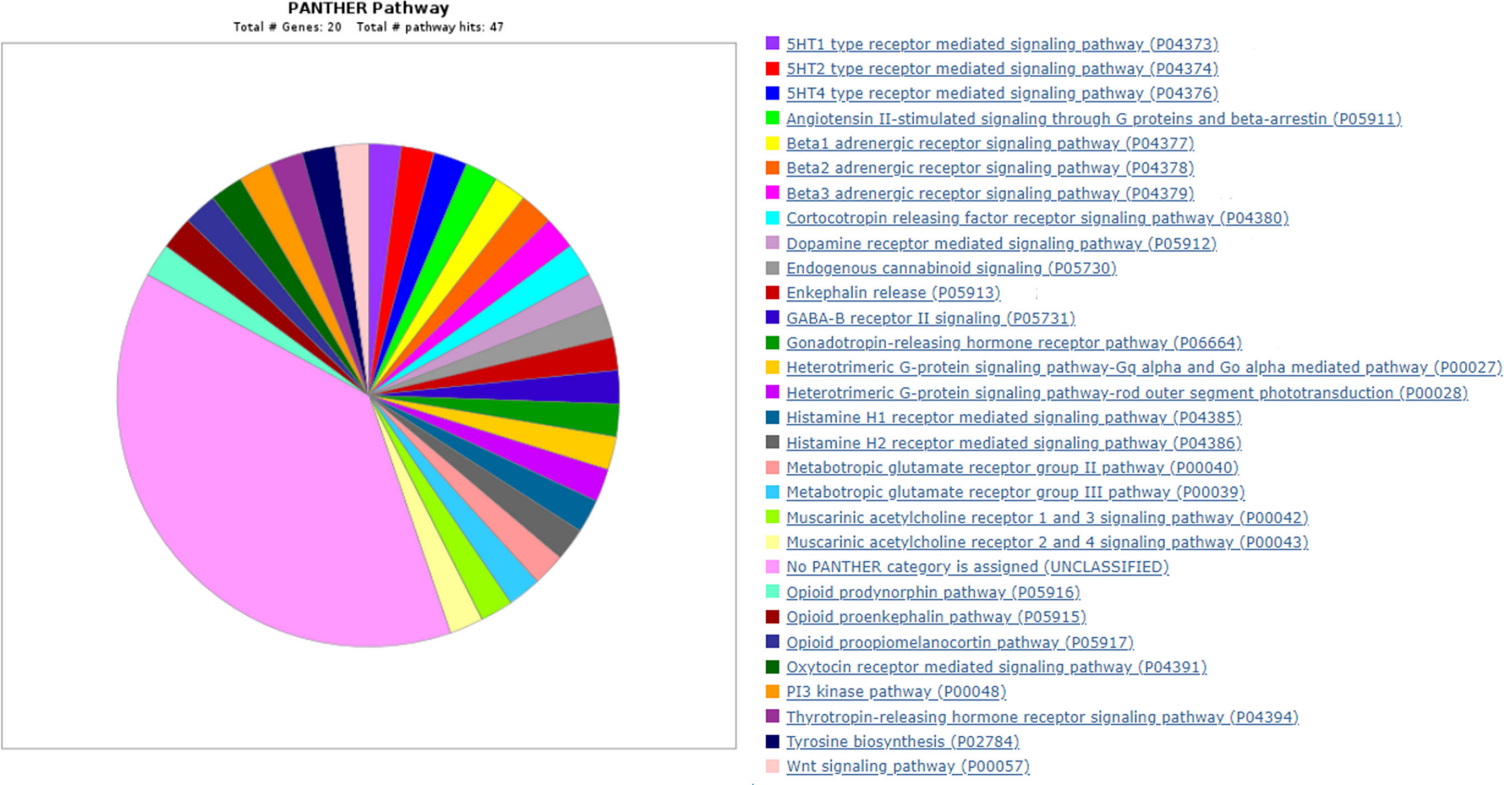
Molecular pathways of 20 candidate genes using the PANTHER tool, with each slice representing the proportion of the specific molecular pathway among the 20 genes.

To determine which molecular pathways were involved in the 20 genes in the human ortholog found set, we used the PANTHER and KEGG bioinformatics tools. PANTHER analysis revealed that many of the genes had no assigned category, whereas the remaining genes were involved in a wide array of pathways, such as the PI3 kinase pathway and Wnt signaling pathway (Fig. 4). Notably, of the eight novel mouse genes corresponding to seven human orthologs with no prior retinal disease association, none had annotations in PANTHER for known pathways. An additional literature review was conducted to evaluate known functions or pathways of these genes. *Atp8b1* is involved in the transfer of phospholipids, with mutations known to cause congenital cholestasis and hearing loss.^21^ Other functions of the novel genes include regulation of N-Ras palmitate turnover (*Abhd17b*),^22^ autophagy of the endoplasmic reticulum and Golgi (*Calcoco1*),^23^ and neuronal apoptosis (*Trim39*).^24^ Some genes have also been implicated in various disease processes, such as chronic obstructive pulmonary disease (*Nt5dc1*),^25^ membranous nephropathy,^26^ and laryngeal squamous cell carcinoma.^27^

KEGG Mapper was also used to identify molecular pathways in which the knockout genes are involved. A wide variety of pathways were identified in which various genes are known to participate, but no single pathway included more than one gene from the human ortholog found set. When KEGG Mapper was used to assess the human ortholog found set in the context of known IRD genes from RetNet, several overlapping pathways were identified: apelin signaling, cholinergic synapse, glutamatergic synapse, GABAergic synapse, pathways in cancer, Ras signaling pathway, chemokine signaling pathway, Kaposi sarcoma-associated herpesvirus infection, retrograde endocannabinoid signaling, PI3K/Akt pathway, and cytomegalovirus infection.

## Discussion

The genetics of retinal biology is a complex area of research. As a result of this complexity, our understanding of the genetic causes of retinal pathology is incomplete, particularly in slow-onset retinal diseases and for diseases of aging. A large number of unique variants and alleles are implicated in retinal diseases. This is further complicated by heterogeneous phenotypes, diverse inheritance patterns, and overlapping causative genes.^28^ An estimated one out of three people worldwide carries at least one pathologic mutation in an established retinal disease gene.^29^^,^^30^ Identification of the causative genes involved is crucial for prognostication and to advance gene-specific therapies, as demonstrated by the success of gene therapy for *RPE65* Leber's congenital amaurosis.^31^

Late-onset IRDs present an additional challenge, as non-specific or heterogeneous visual symptoms and clinical findings may lead to delayed or incorrect diagnoses and cause confusion with more common diagnoses such as AMD. For example, late-onset retinal degeneration is an autosomal dominant IRD that has several features, including subretinal deposits and choroidal neovascularization, that can mimic AMD.

In contrast, AMD is the most common cause of irreversible blindness in the developed world, and it is projected that close to 300 million people worldwide will be affected by 2040.^32^ In contrast to monogenic IRDs, AMD has a multifactorial etiology, including age, genetics, and environmental factors. Genes associated with members of the complement cascade (C2, C3, CFB, and CFH), as well as extracellular matrix regulation and mitochondrial oxidative stress, have been implicated in AMD pathogenesis through GWAS studies.^33^ This has led to the exploration and approval of recent AMD drugs avacincaptad pegol (iZERVAY; Iveric Bio, Parsippany, NJ, USA) and pegcetacoplan (SYFOVRE, Apellis Pharmaceuticals, Waltham, MA, USA), both of which inhibit the complement system and slow progression of macular atrophy. These examples emphasize how genetic understanding of retinal pathobiology leads to development of therapeutic medications that prevent vision loss.

The majority of the genes identified in this study were expressed in the retina and retinal pigment epithelium. However, a few of the genes were not expressed in either of these tissues. This suggests that some gene mutations may have downstream effects in other tissues. For example, *TAT* is primarily expressed in the liver, but a deficiency in *TAT* leads to tyrosinemia, which has systemic and ocular effects due to the accumulation of tyrosine and its byproducts. However, there are no known retinal findings associated with tyrosinemia reported in the literature.

This study is limited in its ability to identify potential biological pathways implicated in AMD and late-onset IRDs. The IMPC late-adult pipeline comprises only about 5% of the knockout mice, and the criteria for selected genes varied across each IMPC center, sometimes based on prior GWAS data, early adult data, or other interests unrelated to the eye. This is relevant, as most IRDs are non-syndromic and are isolated to retina-specific genes.^28^ If more data become available, they may reveal yet to be discovered pathways relevant to the retina. Furthermore, Although the IMPC phenotyping process assesses retinal morphology, confirmatory molecular biological testing in retina or any other tissue, as well as routine histopathology, electroretinography, and optomotor testing to measure retinal/visual function, are beyond the scope of the Consortium.

An additional limitation is the confounding influence of the homozygous *rd8* alleles (*Crb1* gene) in the background of these knockout mice (C57BL/6N background), which causes its own slow retinal degeneration. This *rd8* mutation may result in late-adult retinal phenotypic findings hits that could be a result of interaction between the *Crb1* gene and the targeted deletion.

This study demonstrated relationships between known AMD-associated genes and established IRD genes for several of the 20 human orthologs in the found set. Although mouse models have been useful in understanding the pathophysiology of retinal degenerations such as in AMD,^34^^–^^37^ there are important species-specific differences between mouse and human eye biology. Unlike in the human retina, the mouse retina lacks a macula, and there are species differences in photoreceptor density in the central retina.^38^ The pathologies identified in this study were also primarily retinal in nature, whereas the pathogenesis of AMD appears to originate from the RPE–Bruch's membrane complex. Also, given that C57BL/6 mice have a life expectancy of approximately 120 weeks, the 49- to 80-week “late adult” stage in mice used in this study corresponds to an age of roughly 32 to 52 years in humans, but human AMD typically begins at around age 50 years and becomes severe around age 70. Therefore, the age interval studied may not be ideal to detect AMD-related genes. Additionally, vivarium lighting conditions in mouse facilities are generally lower than those in human daily life, leading to a lower cumulative exposure to damaging ultraviolet light. Furthermore, the absolute age of photoreceptors in aged mice and aged humans differs significantly, which may impact the genesis of AMD. There may also be differences in the aging process in the retina of mice versus primates, such as the degree of inflammation, photoreceptor loss and decrease in ATP production with age^39^; this suggests that genetic mutations may play differing roles in the pathophysiology of aging in the retina between species.

In summary, the 12 mouse genes (corresponding to 20 human orthologs) with significant late-adult abnormal retinal pathology may prove to be relevant in human retinal disease. Eight of these genes have not been previously implicated in retinal physiology or pathology in either mice or humans, making them exciting avenues for further research. The pathways in which these candidate genes are involved may provide new insights into the pathophysiology of slow-onset retinal diseases and help inform the direction of future therapies. These pathways may include the beta-adrenergic receptor,^40^ dopaminergic receptor,^41^ and histamine receptor^42^ mediated signaling, as demonstrated in this study. Additionally, because several of the novel genes have limited data regarding their biological function and pathways, they may represent important yet to be-discovered pathways and functions in the retina. Given that a significant proportion of presumed IRD patients do not receive a molecular diagnosis using currently available tools, we advocate including these candidate genes in IRD genetic testing panels to improve diagnostic rates.

## Supplementary Material

Supplement 1

Supplement 2

Supplement 3

Supplement 4

## Appendix Appendix. The International Mouse Phenotyping Consortium (IMPC)

The following authors are IMPC members who contributed to this study: Elif Acar, Antonio Aguilar-Pimentel, Elizabeth Axton, Shinya Ayabe, Abdel Ayadi, Lore Becker, Alexandr Bezginov, Marie-Christine Birling, Jin Woong Bok, Vivian Bradaschia, Julia Calzada-Wack, Adam Caulder, Linda Chan, Audrey E. Christiansen, Dave Clary, James Cleak, Gemma Codner, Amie Creighton, Patricia da Silva Buttkus, Nathalia Dragano, Kyle Duffin, Qing Fan-Lan, Martin Fray, Tamio Furuse, Xiang Gao, Wendy Gardiner, Lillian Garrett, Angelina Gaspero, Marina Gertsenstein, Isabelle Goncalves, Leslie Goodwin, Kristin Nicole Grimsrud, Alain Guimond, Sabine Hölter-Koch, Joanne H. Hsu, Mizuho Iwama, Lois Kelsey, Chang-Hoon Kim, Kyoungmi Kim, Markus Kraiger, Tatsuya Kushida, Denise Lanza, Valerie Laurin, Sophie Leblanc, Ho Lee, Christoph Lengger, Stefanie Leuchtenberger, Lauri G. Lintott, Jimmy Liu, Yang Liu, Isabel Lorenzo, Aline Lux-Simonet, Susan Marschall, Matthew McKay, Matthew McKenzie, David Miller, Ikuo Miura, Saori Mizuno, Toshiaki Nakashiba, Ki Taek Nam, Clare Norris, Yuichi Obata, Manuela Österreicher, Kristina Palmer, Guillaume Pavlovic, Kevin Peterson, Dawei Qu, Tara Rasmussen, Birgit Rathkolb, Kyle Roberton, Adrian Sanz Moreno, Claudia Seisenberger, Audrie Seluke, Xueyuan Shang, Hirotoshi Shibuya, Gillian Sleep, Nadine Spielmann, Claudia Stöger, Toyoyuki Takada, Nobuhiko Tanaka, Lydia Teboul, Todd Tolentino, Igor Vukobradovic, Hongyu Wang, Brandon Willis, Joshua Wood, Xia Xue Catherine Xu, Ikuko Yamada, Jing Zhao.

## References

[1] Bessant DA, Ali RR, Bhattacharya SS. Molecular genetics and prospects for therapy of the inherited retinal dystrophies. *Curr Opin Genet Dev*. 2001; 11: 307–316. 11377968 10.1016/s0959-437x(00)00195-7

[2] Berger W, Kloeckener-Gruissem B, Neidhardt J. The molecular basis of human retinal and vitreoretinal diseases. *Prog Retin Eye Res*. 2010; 29: 335–375. 20362068 10.1016/j.preteyeres.2010.03.004

[3] Pontikos N, Arno G, Jurkute N, et al. Genetic basis of inherited retinal disease in a molecularly characterized cohort of more Than 3000 families from the United Kingdom. *Ophthalmology*. 2020; 127: 1384–1394. 32423767 10.1016/j.ophtha.2020.04.008 PMC7520514

[4] Goetz KE, Reeves MJ, Gagadam S, et al. Genetic testing for inherited eye conditions in over 6,000 individuals through the eyeGENE network. *Am J Med Genet C Semin Med Genet*. 2020; 184: 828–837. 32893963 10.1002/ajmg.c.31843 PMC8162059

[5] Mustafi D, Hisama FM, Huey J, Chao JR. The current state of genetic testing platforms for inherited retinal diseases. *Ophthalmol Retina*. 2022; 6: 702–710. 35307606 10.1016/j.oret.2022.03.011 PMC9356993

[6] Weisschuh N, Mayer AK, Strom TM, et al. Mutation detection in patients with retinal dystrophies using targeted next generation sequencing. *PLoS One*. 2016; 11: e0145951. 26766544 10.1371/journal.pone.0145951 PMC4713063

[7] Weisschuh N, Obermaier CD, Battke F, et al. Genetic architecture of inherited retinal degeneration in Germany: a large cohort study from a single diagnostic center over a 9-year period. *Hum Mutat*. 2020; 41: 1514–1527. 32531858 10.1002/humu.24064

[8] Ring N, Meehan TF, Blake A, et al. A mouse informatics platform for phenotypic and translational discovery. *Mamm Genome*. 2015; 26: 413–421. 26314589 10.1007/s00335-015-9599-2 PMC4602054

[9] Groza T, Gomez FL, Mashhadi HH, et al. The International Mouse Phenotyping Consortium: comprehensive knockout phenotyping underpinning the study of human disease. *Nucleic Acids Res*. 2023; 51: D1038–D1045. 36305825 10.1093/nar/gkac972 PMC9825559

[10] Karp NA, Meehan TF, Morgan H, et al. Applying the ARRIVE guidelines to an in vivo database. *PLoS Biol*. 2015; 13: e1002151. 25992600 10.1371/journal.pbio.1002151 PMC4439173

[11] Swamy VS, Fufa TD, Hufnagel RB, McGaughey DM. Building the mega single-cell transcriptome ocular meta-atlas. *GigaScience*. 2021; 10: giab061. 34651173 10.1093/gigascience/giab061 PMC8514335

[12] Moore BA, Leonard BC, Sebbag L, et al. Identification of genes required for eye development by high-throughput screening of mouse knockouts. *Commun Biol*. 2018; 1: 236. 30588515 10.1038/s42003-018-0226-0 PMC6303268

[13] Mattapallil MJ, Wawrousek EF, Chan CC, et al. The Rd8 mutation of the Crb1 gene is present in vendor lines of C57BL/6N mice and embryonic stem cells, and confounds ocular induced mutant phenotypes. *Invest Ophthalmol Vis Sci*. 2012; 53: 2921–2927. 22447858 10.1167/iovs.12-9662 PMC3376073

[14] Moore BA, Roux MJ, Sebbag L, et al. A population study of common ocular abnormalities in C57BL/6N rd8 mice. *Invest Ophthalmol Vis Sci*. 2018; 59: 2252–2261. 29847629 10.1167/iovs.17-23513 PMC5935295

[15] Shiba T, Bujo H, Takahashi M, et al. Vitreous fluid and circulating levels of soluble lr11, a novel marker for progression of diabetic retinopathy. *Graefes Arch Clin Exp Ophthalmol*. 2013; 251: 2689–2695. 23652469 10.1007/s00417-013-2373-9

[16] García M, Álvarez L, Fernández Á, et al. Metallothionein polymorphisms in a Northern Spanish population with neovascular and dry forms of age-related macular degeneration. *Ophthalmic Genet*. 2017; 38: 451–458. 28635422 10.1080/13816810.2017.1288825

[17] Saddala MS, Lennikov A, Grab DJ, Liu GS, Tang S, Huang H. Proteomics reveals ablation of PlGF increases antioxidant and neuroprotective proteins in the diabetic mouse retina. *Sci Rep*. 2018; 8: 16728. 30425286 10.1038/s41598-018-34955-x PMC6233167

[18] Monti G, Jensen ML, Mehmedbasic A, et al. SORLA expression in synaptic plexiform layers of mouse retina. *Mol Neurobiol*. 2020; 57: 3106–3117. 32472518 10.1007/s12035-020-01946-x

[19] Gibson CJ . Alterations in retinal tyrosine and dopamine levels in rats consuming protein or tyrosine-supplemented diets. *J Neurochem*. 1988; 50: 1769–1774. 2897426 10.1111/j.1471-4159.1988.tb02477.x

[20] Alvarez-Mora MI, Blanco-Palmero VA, Quesada-Espinosa JF, et al. Heterozygous and homozygous variants in SORL1 gene in Alzheimer's disease patients: clinical, neuroimaging and neuropathological findings. *Int J Mol Sci*. 2022; 23: 4230. 35457051 10.3390/ijms23084230 PMC9024679

[21] Korneenko TV, Pestov NB, Okkelman IA, Modyanov NN, Shakhparonov MI. [P4-ATP-ase Atp8b1/FIC1: structural properties and (patho)physiological functions]. *Bioorg Khim*. 2015; 41: 3–12. 26050466 10.1134/s1068162015010070

[22] Lin DT, Conibear E. ABHD17 proteins are novel protein depalmitoylases that regulate N-Ras palmitate turnover and subcellular localization. *eLife*. 2015; 4: e11306. 26701913 10.7554/eLife.11306 PMC4755737

[23] Nthiga TM, Kumar Shrestha B, Lamark T, Johansen T. The soluble reticulophagy receptor CALCOCO1 is also a Golgiphagy receptor. *Autophagy*. 2021; 17: 2051–2052. 34162311 10.1080/15548627.2021.1940610 PMC8386724

[24] Basu-Shrivastava M, Mojsa B, Mora S, et al. Trim39 regulates neuronal apoptosis by acting as a SUMO-targeted E3 ubiquitin-ligase for the transcription factor NFATc3. *Cell Death Differ*. 2022; 29: 2107–2122. 35449213 10.1038/s41418-022-01002-2 PMC9613758

[25] Guo Y, Gong Y, Shi G, et al. Single-nucleotide polymorphisms in the TSPYL-4 and NT5DC1 genes are associated with susceptibility to chronic obstructive pulmonary disease. *Mol Med Rep*. 2012; 6: 631–638. 22736055 10.3892/mmr.2012.964

[26] Jiang S, Jiang D, Lian Z, Huang X, Li T, Zhang Y. THSD7A as a promising biomarker for membranous nephrosis. *Mol Biotechnol*. 2024; 66: 3117–3135. 37884765 10.1007/s12033-023-00934-5

[27] Hui L, Wang J, Zhang J, Long J. lncRNA TMEM51-AS1 and RUSC1-AS1 function as ceRNAs for induction of laryngeal squamous cell carcinoma and prediction of prognosis. *PeerJ*. 2019; 7: e7456. 31565549 10.7717/peerj.7456 PMC6743450

[28] Schneider N, Sundaresan Y, Gopalakrishnan P, et al. Inherited retinal diseases: linking genes, disease-causing variants, and relevant therapeutic modalities. *Prog Retin Eye Res*. 2022; 89: 101029. 34839010 10.1016/j.preteyeres.2021.101029

[29] Hanany M, Rivolta C, Sharon D. Worldwide carrier frequency and genetic prevalence of autosomal recessive inherited retinal diseases. *Proc Natl Acad Sci USA*. 2020; 117: 2710–2716. 31964843 10.1073/pnas.1913179117 PMC7007541

[30] Rivolta C, Sharon D, DeAngelis MM, Dryja TP. Retinitis pigmentosa and allied diseases: numerous diseases, genes, and inheritance patterns. *Hum Mol Genet*. 2002; 11: 1219–1227. 12015282 10.1093/hmg/11.10.1219

[31] Fenner BJ, Tan TE, Barathi AV, et al. Gene-based therapeutics for inherited retinal diseases. *Front Genet*. 2021; 12: 794805. 35069693 10.3389/fgene.2021.794805 PMC8782148

[32] Vyawahare H, Shinde P. Age-related macular degeneration: epidemiology, pathophysiology, diagnosis, and treatment. *Cureus*. 2022; 14: e29583. 36312607 10.7759/cureus.29583 PMC9595233

[33] Katta S, Kaur I, Chakrabarti S. The molecular genetic basis of age-related macular degeneration: an overview. *J Genet*. 2009; 88: 425–449. 20090206 10.1007/s12041-009-0064-4

[34] Rakoczy PE, Zhang D, Robertson T, et al. Progressive age-related changes similar to age-related macular degeneration in a transgenic mouse model. *Am J Pathol*. 2002; 161: 1515–1524. 12368224 10.1016/S0002-9440(10)64427-6 PMC1867306

[35] Espinosa-Heidmann DG, Suner IJ, Catanuto P, Hernandez EP, Marin-Castano ME, Cousins SW. Cigarette smoke-related oxidants and the development of sub-RPE deposits in an experimental animal model of dry AMD. *Invest Ophthalmol Vis Sci*. 2006; 47: 729–737. 16431974 10.1167/iovs.05-0719

[36] Coffey PJ, Gias C, McDermott CJ, et al. Complement factor H deficiency in aged mice causes retinal abnormalities and visual dysfunction. *Proc Natl Acad Sci USA*. 2007; 104: 16651–16656. 17921253 10.1073/pnas.0705079104 PMC2034255

[37] Marmorstein LY, McLaughlin PJ, Peachey NS, Sasaki T, Marmorstein AD. Formation and progression of sub-retinal pigment epithelium deposits in Efemp1 mutation knock-in mice: a model for the early pathogenic course of macular degeneration. *Hum Mol Genet*. 2007; 16: 2423–2432. 17664227 10.1093/hmg/ddm199

[38] Volland S, Esteve-Rudd J, Hoo J, Yee C, Williams DS. A comparison of some organizational characteristics of the mouse central retina and the human macula. *PLoS One*. 2015; 10: e0125631. 25923208 10.1371/journal.pone.0125631 PMC4414478

[39] Kam JH, Weinrich TW, Shinhmar H, et al. Fundamental differences in patterns of retinal ageing between primates and mice. *Sci Rep*. 2019; 9: 12574. 31467395 10.1038/s41598-019-49121-0 PMC6715671

[40] Ruan Y, Böhmer T, Jiang S, Gericke A. The role of adrenoceptors in the retina. *Cells*. 2020; 9: 2594. 33287335 10.3390/cells9122594 PMC7761662

[41] Witkovsky P . Dopamine and retinal function. *Doc Ophthalmol*. 2004; 108: 17–40. 15104164 10.1023/b:doop.0000019487.88486.0a

[42] Gastinger MJ, Barber AJ, Vardi N, Marshak DW. Histamine receptors in mammalian retinas. *J Comp Neurol*. 2006; 495: 658–667. 16506196 10.1002/cne.20902 PMC3348866

