# Supplementary figures and images for: Ocular Phenotyping of Knockout Mice Identifies Genes Associated With Late Adult Retinal Phenotypes
Source: Invest Ophthalmol Vis Sci. 2025 Jun 23;66(6):64. doi: 10.1167/iovs.66.6.64 (PMC12186831; doi:10.1167/iovs.66.6.64)

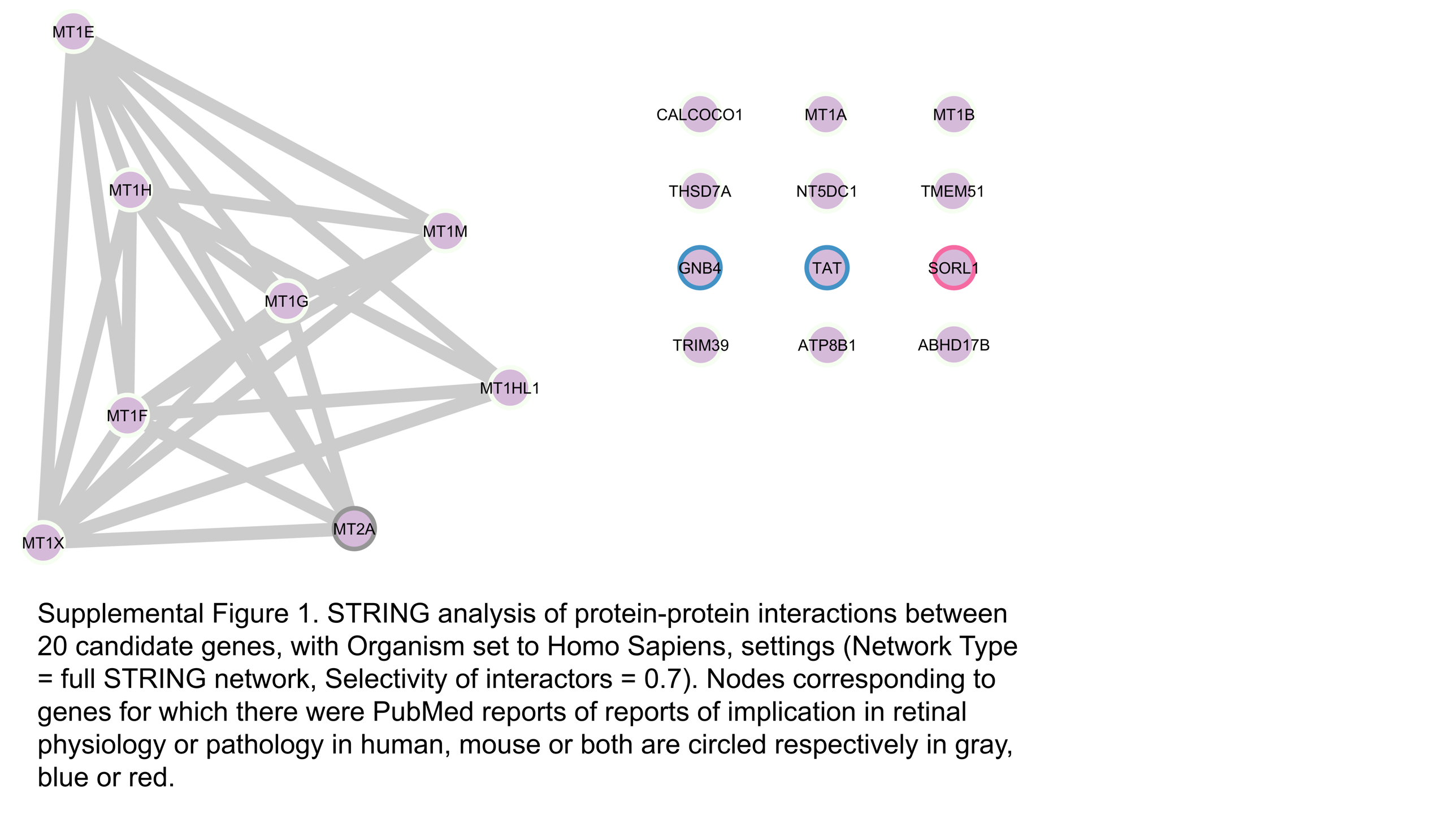

Supplement: Supplement 1 [file iovs-66-6-64_s001.gif]

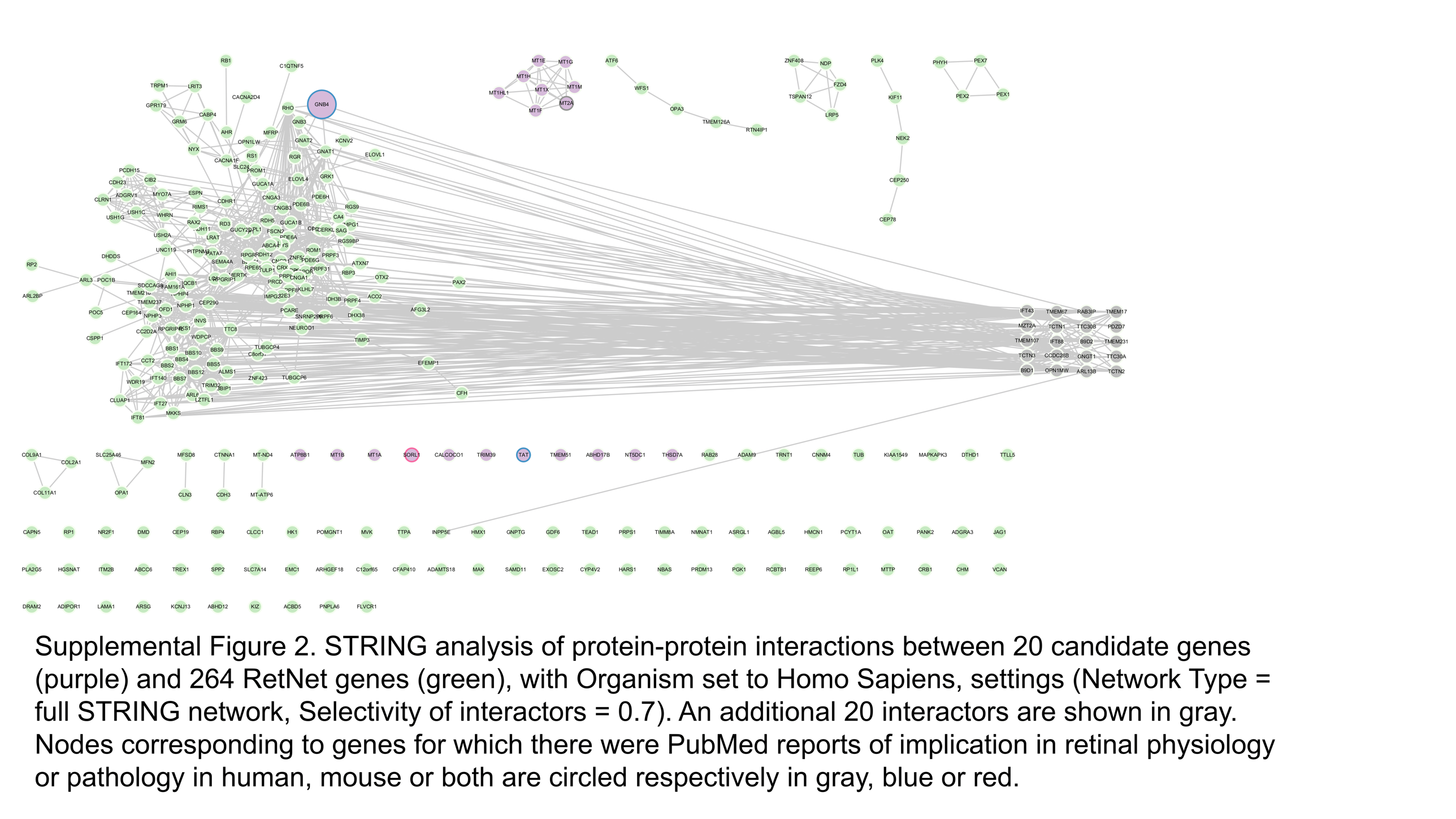

Supplement: Supplement 2 [file iovs-66-6-64_s002.gif]

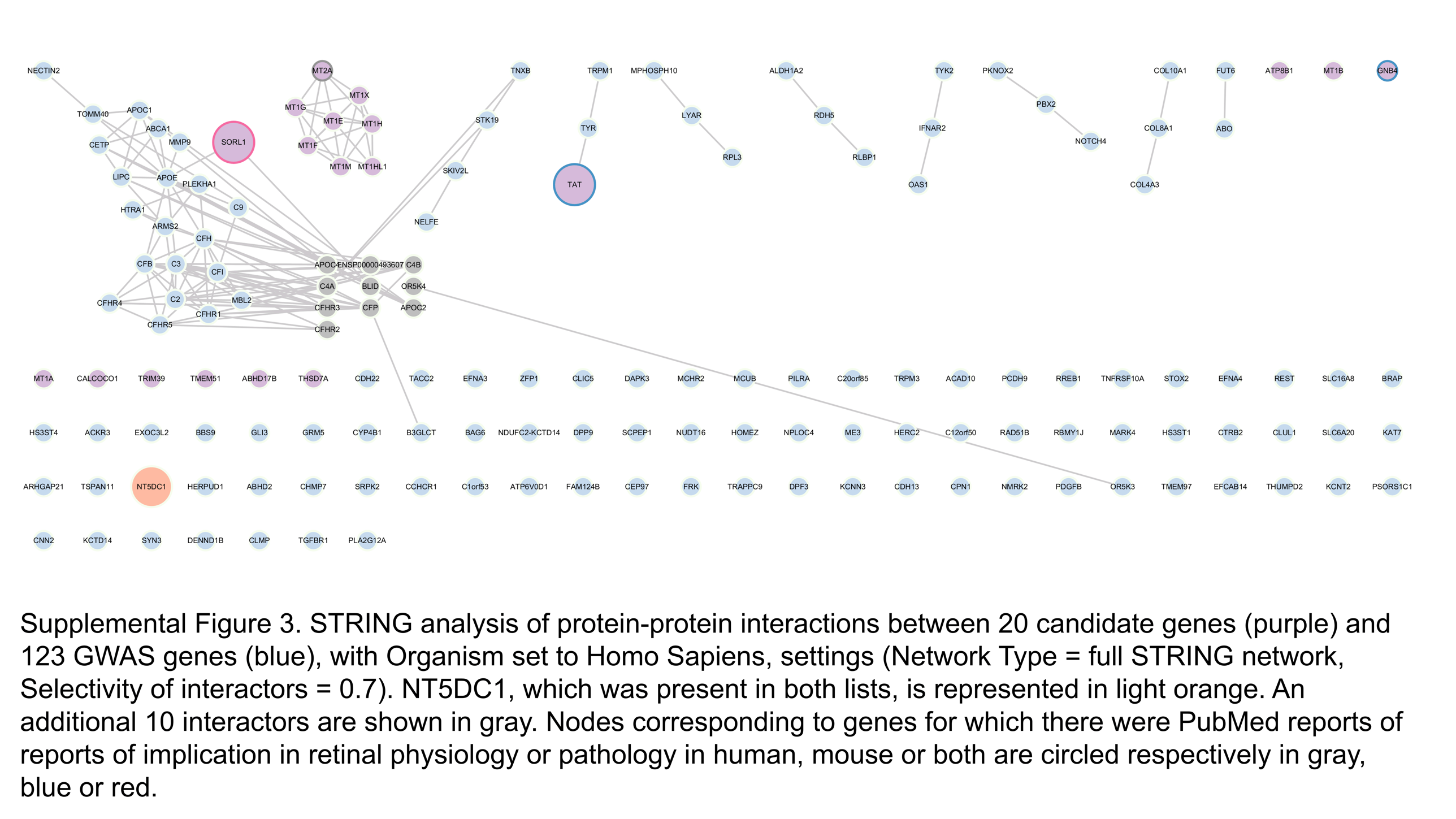

Supplement: Supplement 3 [file iovs-66-6-64_s003.gif]
